# Policy analysis of salt reduction in bread in Iran

**DOI:** 10.3934/publichealth.2019.4.534

**Published:** 2019-12-09

**Authors:** Saba Loloei, Hamed Pouraram, Reza Majdzadeh, Amirhossein Takian, Massomeh Goshtaei, Abolghasem Djazayery

**Affiliations:** 1 Department of Community Nutrition, School of Nutritional Sciences and Dietetics, Tehran University of Medical Sciences, Tehran, Iran; 2 Department of Epidemiology and Biostatistics, School of Public Health, Tehran University of Medical Sciences, Tehran, Iran; 3 Department of Health Services Management and Economics, School of Public Health, Tehran University of Medical Sciences, Tehran, Iran; 4 Health Equity Research Center, Tehran University of Medical Sciences, Tehran, Iran; 5 Iran University of Medical Sciences, Tehran, Iran

**Keywords:** policy analysis, policy context, policy content, policy process, actors, salt reduction

## Abstract

Given that average salt intake among Iranians is approximately 10–15 g per day particularly from sodium hidden in bread, cheese, and fast food; lowering this mineral has been followed up seriously in this country for almost 10 years (since 2009). The main objective of the present study was to provide an opportunity to recognize unwanted and unfavorable outcomes of implementing decisions and policies together with associated problems of salt reduction in bread in order to achieve national and global health promotion goals. Thus, this qualitative and retrospective policy analysis was completed to evaluate the policy of salt reduction in bread in Iran. To collect the data, the researchers traveled to six cities in different regions, wherein relevant documents were utilized added to interviews with key actors. Related websites were correspondingly searched to find reports on this policy. Moreover, the researchers referred to some organizations in-person to search documents in this area. Five group discussions were also held to obtain public opinions in this regard. Data analysis was further carried out using framework analysis. The findings revealed that allocation of the highest rates of subsidy to wheat, flour, and bread had led to elimination of competitiveness in wheat, flour, and bread supply chain in Iran. Despite the presence of proper structures as coordinators of other organizations working on public health, there was no intersectoral collaboration in terms of maintaining health of bread products and lowering salt content in this staple food. With regard to changes in priorities of the Iranian Ministry of Health and Medical Education, attempts made to improve bread quality had also failed. In addition, first-line staff (i.e. bakers) had viewed formulation and implementation of the given policy as a top-down one. Given the ambiguities in establishment of new standards, there were similarly contradictions in execution of the policy at various levels. With reference to education provided at a national level, it was concluded that some people had become more sensitive to salt reduction in bread to some extent.

## 1. Introduction

After Turkey, Iranians consume the most bread per capita in the world in a way that bread consumption rate in Iran is almost twice as those in European countries [1]. Bread is categorized as a staple food worldwide [2],[3], being responsible for an average of 30% of daily salt intake [4],[5]. It is also recognized as the first food among Iranians, whose daily consumption is very common [6]. Accordingly; salt plays an important role in qualitative properties of bread including giving flavor to it, controlling yeast growth and fermentation, improving product texture, as well as reducing bread mold especially spoilage [7]. While adding salt to bread is inappropriate in terms of nutritional principles, it can technologically have positive effects at every stage of production including mixing, fermentation, and baking, as well as characteristics of the final product [8],[9]. High salt intake is associated not only with hypertension but also with some vascular anomalies that may increase independently [8]. Furthermore, sodium intake causes arterial stiffness independently of hypertension [10] and can be associated with diseases such as gastric cancer [11] and osteoporosis [12].

Among the countries reviewed in the national action plan for non-communicable disease (NCD) prevention and control by the National and Sub-National Burden of Diseases Study (NASBOD), Iran has the highest rates of mortality and disability-adjusted life year (DALY) due to high salt intake [13]. Recently, health policy institutions have been also planning to reduce the amount of sodium consumed by communities due to increased costs in health systems and an elevated number of patients affected with cardiovascular diseases (CVDs) [7]. In this respect, the World Health Organization (WHO) identifies salt reduction as the “best choice” in terms of cost-effectiveness and feasibility of this approach in prevention of NCDs [14]. Accordingly, it has been estimated that rates of strokes and CVDs will decrease by 23% and 17%, respectively, as the daily salt intake is reduced from 10 g per day to 5 g [15].

In addition, reducing the amount of salt intake to 6 g per day can prevent 2.5 million deaths each year across the world [15]. Lowering sodium intake has been followed up seriously by the Iranian Ministry of Health and Medical Education and all its subsidiary units over the past 10 years (since 2009) [15]. Given the fact that Iranians consume 15–10 g of salt every day [15], with a large percentage related to bread, cheese, and fast food [16]; various policies have been developed to cut salt in bread as a staple food.

It should be noted that reduction in salt levels in bread affects several qualitative characteristics of this product, which are of importance in terms of customer acceptance and industry [7]. In addition, bakers are reluctant to lower salt content since they believe that it might change bread taste and quality. Furthermore, they are not likely to abide by these strategies due to performing longer mixing and dough-processing periods [18]. Accordingly, assessment of such policies along with stakeholder analysis might result in recognition of unintended and inappropriate outcomes arising from implementation of decisions and policies as well as their related barriers to achieving national goals to promote health and to moderate prevalence and occurrence of NCDs, to help formulate and implement these policies, and to utilize technical evidence in support of policy processes [18]. The main objective of this study was to provide an opportunity to recognize the unwanted and unfavorable outcomes of implementing decisions and policies as well as associated problems of salt reduction in bread in order to accomplish national and global health promotion goals.

## 2. Materials and methods

This study was a qualitative and retrospective policy analysis, in which policy-making for reduction of salt in bread in Iran in 2018 was described and assessed. As a general rule, qualitative studies are appropriate for illustrating and analyzing issues that have not been thoroughly investigated until now. In addition, these types of studies provide comprehensive and complete data on the subject matter [20].

In the present study, three data collection methods were utilized including interview, observation, and focus group discussion.

### 2.1. Interview

The study population consisted of a total of 37 informants and key individuals including two groups of administrative assistants and experts in policy-making for salt reduction in bread such as university professors with a history of research in this field, managers, as well as policy-makers and specialists with an administrative experience regarding salt and bread policies (Box 1), selected via snowball sampling as a purposeful method of sampling. The data were then collected through semi-structured interviews with the participants in a face-to-face manner. This research tool encompassed open-ended questions prepared by the interviewer (thematic interview guide) and also created naturally throughout the interview. It is noteworthy that the 15-item thematic interview guide on salt reduction in bread (Appendix 1) was developed based on the researchers' experiences and evaluations of the related literature. In total, five pilot interviews were completed prior to the study.

A consent form was further obtained from the interviewees, in-person or through a phone call. Afterwards; the research characteristics sheet was submitted to the participants, in-person or through emails, and they were returned after completion. Each interview lasted about 45 minutes and the data were collected during six months. In order to record the interviewees' voices, they were ensured of confidentiality of the data and their personal information. No consent to record their voices was also resolved by taking notes by the interviewer. The audio files were subsequently transcribed and a copy was then sent to the interviewees to confirm the accuracy of the data in case of any doubt in their content. The interviews also continued until data saturation.

It should be noted that the researchers traveled to six different parts of Iran, and used relevant documents along with interviews with key actors. These cities were selected mainly based on the quality and quantity of wheat produced. There were also attempts to cover the entire country geographically.

This research was approved by the Research Ethics Committee of Tehran University of Medical Sciences, Iran.

Box 1. **Interviewees.**

**At the national level:**

- Iranian Ministry of Health and Medical Education: 1 former head of the office and 1 head of the current Office of Social Nutrition Improvement, 1 major policy-maker, and 3 supervisors
- Ministry of Agriculture Jihad: 1 individual in monitoring units
- Tehran Bakers Union: 1 individual
- Iran's Flour Industries Union: 1 individual
- Iranian National Standards Organization: 1 individual

**At the provincial level:**

- Environmental health inspectors: 5 individuals
- Government Trading Corporation of Iran: 5 individuals
- Ministry of Agriculture Jihad: 2 individuals
- University professors: 5 individuals
- Bakers (industrial and traditional): 10 individuals

### 2.2. Observation

The researcher attended bakeries (traditional and industrial) as well as traditional flour mills and assessed their work method, especially use of salt in bread. If needed, some processes were photographed and videotaped after receiving permissions. Other resources exploited in this study were documents and reports. To this end; the websites of the WHO, as well as the domestic ones were searched to find reports related to the policy of salt reduction in bread in Iran. Furthermore, the researchers referred to some organizations in-person to search for relevant documents. Other documents utilized included articles, newspapers, magazines, authorized news agency websites, as well as annual and six-month reports from various centers. The documents were also used as additional resources for validating other collected data.

### 2.3. Focus group discussion

A total of five focus group discussions were held while people were waiting in the queue of bakeries, to obtain public opinions in this respect and to gather information about the effects of the policy of salt reduction in bread on people.

## 3. Data analysis

In the vein of most qualitative studies, data analysis was performed simultaneously with data collection. This concomitance allows for returning to questions and correcting, modifying, or eliminating them for future interviews. It should be noted that the data were analyzed using framework analysis, formed based on existing ideas (i.e. a predetermined framework) which does not seek to develop a new theory. In the present study, the framework employed was policy analysis triangle adapted from Walt and Gilson (1994), encompassing four categories of context, actors, process, and content in the analysis of policies (20).

Credibility was also measured to ensure the accuracy of the results. In order to boost credibility, participants with sufficient experience in this area were selected and then integration was applied in all stages of the study including diversity in data collection methods (methodological triangulation) (e.g., interview, observation, and focus group discussion). Moreover, there were attempts to allot sufficient time to data analysis. If required, the transcribed interviews were returned to the interviewees for further revisions. To improve transferability, all research stages were recorded to allow for the follow-up of each stage and to have a clear process. In addition to citations, research design and stages were explicated. Given the fact that confirmability is interpreted as unbiased data, controversial and negative cases were evaluated to understand the reason for inconsistency between results. Furthermore, long-term presence of the researcher and allocation of sufficient time, accuracy in all research stages, and clarity of the research methodology could help concretize the data. In addition, dependency was obtained using complementary views of supervisors and counselors in terms of coding and analyzing the transcribed interviews.

## 4. Results

The results were reported in four categories of the policy analysis triangle framework i.e. context, policy content, policy-making process, and actors.

### 4.1. Context

Implementation of a new policy depends on the setting in which it is formed. In this part of the study, the environmental aspects having direct or indirect effects on the policy of salt reduction in bread were delineated. In terms of wheat, it could be concluded that all the relevant stages had been monopolized by the Iranian government with the exception of production. While the state had devoted its main subsidy to wheat, flour, and bread with the aim of protecting vulnerable populations, this act had led to loss of competitiveness in wheat, flour, and bread supply chain in this country. Given the effect of the competitiveness on increasing product quality and the direct relationship between use of salt in bread and quality of wheat and flour; the sovereignty of the government over wheat, flour, and bread was considered as one of the challenges to successful execution of the this policy. For example, one of the interviewees had stated that:

“Salt strengthens gluten bonding and gives it structure. So, relative success will be achieved in implementing this policy if wheat quality is overlooked and I think that it is aimed to solve the problems just in the last stage of the production process (I mean, bakeries)”

(A university professor)

Over recent years, the main government incentive has been allocated to provision, supply, and distribution of wheat and bread. As a result, bread is available to Iranians at a supported cost to protect vulnerable populations. Due to continuous increase in the cost of flour and the constant rate of selling flour to bakeries, bread subsidies have further generated adverse effects such as increased government spending, difference between bread costs in urban and rural areas, as well as bread waste off the scale. Despite much emphasis of high-level documents on supports for bread production industry and promotion of culture of using industrial bread in this country, traditional flat breads, unfortunately, constitute 93% of the bread consumed by the public, and the overall popularity of loaf bread is negligible. In this respect, a former policy-maker had said that:

“In the course of industrial bread production, majority of processes are automatically carried out and less flour is thus wasted. In addition, fermentation is definitely performed and salt use can be properly controlled. Even salt-free bread can be produced. Moreover, bread is baked with indirect heat and there is no residual of fossil fuels on the bread”

(Former nutritional policy-maker)

Generally; in addition to the completely mechanized production process of industrial bread, fermentation is more complete and the final product is endowed with high food safety and health. Owing to their stable production processes and equipment, adhering to bread production standards is thus more practical in these units and they can be effectively monitored. Moreover, monitoring these units and assessing their compliance with the standards can be carried out with high operational capabilities.

In spite of the structure of the Supreme Council of Health and Food Security as the coordinator of other organizations working on public health, all state-run agencies involved in wheat, flour, and bread pursue their own goals and there is less intersectoral collaboration regarding the issues of bread health and salt reduction in this staple food.

Accordingly, three important factors affecting quality and health of the final product (i.e. traditional types of bread) include right ingredients; knowledge, education, and specialty of workforce (that is, bakers), and use of appropriate tools and technologies in bread preparation. The most popular of the traditional Iranian bread prepared in Iran have been already mentioned in [6]. Unfortunately, due to long work hours and difficulty of bakery, especially in hot seasons, skilled workers are reluctant to be in bakeries and they merely work as temporary ones. Accordingly, unwillingness leads to a decrease in effectiveness of vocational training to such workers in bakeries, and ultimately reduces quality and health of the final product.

Bakery equipment is another issue to be considered; since need for salt used in bread is reduced in some cases, according to observations, such as use of dough sheeter machines in *Lavash* flat bread bakeries, because the dough is uniformly spread under the pressure and there is less probability of being torn. Therefore; use of salt, which is mainly to prevent the tearing of bread decreases in the production phase.

### 4.2. Policy content

Following the former order of the Iranian Ministry of Health and Medical Education in 2013, a specialized working group was established to promote bread quality with the main objective of providing effective and practical solutions to eliminate use of baking soda and reducing salt content in bread production process. The intervention recommended included improving formulation of traditional (i.e. flat) bread to provide a model of healthy bread, reviewing list of materials authorized and used in baking industry, revising national standards and guidelines, reducing problems through industrialization of traditional bread production (in other words, changing bread consumption pattern), building proper culture (through Islamic Republic of Iran Broadcasting, newspapers, books, educational booklets, and urban advertising) for healthy bread consumption, determining quality indices of wheat for domestic purchases and imports of wheat in order to take account of effective components in production of high-quality flour and bread products such as gluten, employing food industry graduates to implement precise technical and sanitary supervision on flat and loaf bread bakeries, as well as closing traditional bakeries to build industrial complexes of traditional (flat) breads to improve quality of this product. Unfortunately, attempts made in bread production and results of meetings by the specialized working group on promotion of bread quality have failed due to changes in the government and the priorities of the Iranian Ministry of Health and Medical Education.

### 4.3. Policy-making process

One of the most common and useful methods for evaluating and analyzing the policy-making process is the use of heuristic stages [22]. This model encompasses four stages of agenda-setting, policy formulation, policy implementation, and policy evaluation.

#### 4.3.1. Agenda-setting

Given the much importance of bread along with complexity of wheat, flour, and bread issues; bread quality improvement was put into operation in early 2014. Eventually; after numerous meetings, review of bread and flour standards and monitoring of their proper implementation were announced and approved in 2015. Along with the implementation of the Health Promotion in Iran's five-year Development Plans in the field of prevention and health, environmental health inspectors became equipped with portable salinity meters to measure and record salt content of dough in bakeries.

#### 4.3.2. Policy formulation

Unfortunately, first-line staff (i.e. bakers) has considered the policy of standard reduction of salt in bread as a top-down one, and even impossible in some areas. In the top-down approach, policy-makers are key actors and the role of other stakeholders is overlooked. In this approach, the government is usually recognized as the main ruler, and other actors, including individuals and organizations, are merely subordinates. With regard to a limited perspective in policy-making, there is little chance that all prerequisites for successful policy implementation are taken into account. For example, one of the interviewees had reiterated that:

“The current policy is just an order. Salt in bread should be approximately 1%, but what is the strategy? How can we fulfill it? The imperative policy is not the answer. I think in-depth interviews must be conducted with bakers in order to perceive the problem”

(A provincial environmental health inspector)

In the top-down approach, first-line actors are also dissatisfied with the policy formulated and show little commitment for its implementation, which means failure in policy execution. In addition, some stakeholders had controversial ideas concerning reduction of salt in bread, which seemed to be unknown to main policy-makers in the policy-making process. In this respect, two employees had stated that:

“A big problem is that we ignore salt functions. The salt function is being substituted by some additives whose recognition is not possible due to lack of a proper method. Our major concern is use of unauthorized salt substitutes”

(An employee in Iranian National Standards Organization)

“Memory loss and goiter has increased in this province since the implementation of salt reduction program”

(A provincial employee of Government Trading Corporation of Iran).

#### 4.3.3. Policy implementation

According to Standard No. 2628 of the Institute of Standards and Industrial Research of Iran, salt content in the dry matter of bread should be measured and reported. Meanwhile, environmental health officers need to measure amount of salt in dough using salinity meters and then consider cases above 1% as violation of standard regulations. At the same time, in order to adhere to the standard of 1 g salt per 100 g of bread, bakers should consider a bag of flour as a weight criterion and add 400 g of salt to it (40 kg). It is clear that the standard of salt reduction in bread is not clear and such a contradiction in implementation of the given standard should be resolved as soon as possible.

Furthermore, water quality is of great importance and very hard or soft water is not suitable for baking. In general, water hardness affects dough and the resulting bread, since minerals in water can exert impacts on rising process and solubility, intensity of fermentation, aromatic substances, and bread taste. Very soft water also causes numerous problems due to production of runny dough [23]. In some regions of Iran, the amount of salt and minerals in water used for making dough and baking bread is extremely high. According to bakers, the amount of salt used in bread depends on the season and the temperature of the region. Studies in this line have also demonstrated that salt percentage in traditional bread is lower in the second six months of the year (compliance with the new standard, in 71% of samples), compared with the first six months of the year (compliance with the new standard, in 52% of samples). Moreover; there are many different climates in Iran, so it is not feasible to determine a single standard of salt content in bread for the whole country during a year.

Some people have also questioned the ability to apply the new standard and have further mentioned that the current flour quality does not allow compliance with this standard in Iran. Others have also considered the implementation of this policy to be based on bakers' skills and have expressed that if bakers receive the necessary training from reputable organizations, such as Iran Technical and Vocational Organizations, the given standard can be properly adhered to and bread quality will be significantly improved. Some issues such as lack of attractiveness of bakery job due to its demanding conditions and low wages, seasonal nature of bakery workers in most parts of Iran, and lack of specialized bakery training centers have all led to serious problems in bakery training, especially in reducing salt in traditional bread.

#### 4.3.4. Policy evaluation

The policy of salt reduction in bread officially started at the beginning of 2016 and it has not been so far evaluated in research studies, to the best of authors' knowledge. However, a study conducted in southeastern Iran in 2015 showed that salt reduction in bread could be an acceptable and effective intervention in lowering uric sodium and systolic blood pressure in the community [1].

### 4.4. Actors

#### 4.4.1. Impact of policy of salt reduction in bread on bakers

Some bakers believed that similar pricing by the government regarding both low-quality and high-quality subsidized bread producers had put an end to competitions among bakers to offer a high-quality product. For example, an expert had said that:

“The biggest problem is the subsidy. In fact, we have done away with competitiveness in this industry. This also applies to flour factories. When the government purchases any type of flour, I think there is no point in increasing its quality”

(A provincial health expert)

Others had also assumed that encouragements and incentive policies would be more effective instead of punitive ones together with penalties for bakeries. In this respect, a baker had added that:

“For example, they could put a sign on top of the bakery highlighting that the baker has managed to reduce salt content in bread. This would both encourage bakers and lead to an increase in the number of customers”

(A baker)

#### 4.4.2. Impact of policy of salt reduction in bread on the public

One of the policies adopted by the government is to use the Primary Health Care (PHC) system to educate people about needs for consuming low-salt products. In this way, bread producers (industrial and traditional) will try to offer low-salt bread when the public demands this type of product. The important note is gradual reduction of salt in such products. As seen in other countries such as the United Kingdom, salt reduction must be slow but sure in order to get people to eat low-salt products and to prevent decline in sales [24]. In focus group discussion, one major concern was also iodine deficiency due to beliefs that the only source of iodine was edible salt. Given the greater tangibility of complications of iodine deficiency to these individuals, compared with NCDs, there were serious questions and concerns among them regarding this issue. For example, one of the individuals in the focus group discussion had stated that:

“The public has shown and will show positive attitudes to any factor contributing to their health. Reducing salt intake is no exception, and people are willing to reduce salt content in bread and other products. However, this decrease in salt consumption should be managed in a way that other problems (e.g. iodine deficiency) and other complications are avoided”

Some people have also become so sensitive to lowering salt content in bread, so they would purchase from another bakery in case of detecting a high salt content in bread of a certain bakery. Due to the risks of diseases associated with high salt intake, some people are not just satisfied with reduction of salt content in bread and demand a decrease of this mineral substance in other salty food, including pickles and different types of sauce. On the other hand, some people are indifferent to the amount of salt in bread and only focus on its quality, taste, and appearance, as well as shelf life.

## 5. Discussion

Despite the important role of bread as the main source of salt in the diet of Iranian people, no research has been conducted thus far on reduction of salt content in bread in this country to the best of authors' knowledge. In the present study, the policy of salt reduction in bread was thus evaluated from different viewpoints. While the policy seemed initially simple, its complicated dimensions were recognized after the study and some serious challenges were also found, especially in terms of implementation of the given policy in Iran.

In this regard, the main problem in the area of bread production and not just reduction of salt in this product was lack of competitions either in terms of use of the main raw material, namely flour, or in the final product, i.e. bread types. On the other hand, given the strategic importance of wheat, flour, and bread and the significance of wheat in Iranian households' food basket; governmental supports for producers and consumers, especially vulnerable populations, are inevitable [25]. One of the solutions suggested to eliminate this problem is subsidy allocation of the healthier types of flour (whole-wheat and high-gluten content) and bread (e.g. *Sangak*, because of using whole-wheat flour and thorough fermentation in the process of its production by sourdough starter), so that healthy nutrition in the community can be indirectly promoted and a competitive environment can be created for other types of flour and bread.

Ambiguity in implementing this policy is also due to lack of understanding of the environment, definition of vague goals, as well as no familiarity with existing facilities of organizations [26]. Given the presence of portable salinity meters for monitoring the implementation of this policy, it is suggested that the amount of salt allowed in the dough of traditional bread types be used instead of the permitted salt in the final dry matter in the new standard of salt content in bread. Doing so, contradictions in implementing policies will be removed and then conditions for production of bread types and amount of salt and water used in different types of bread will be taken into account.

Another point is that central policy-makers should not expect unconditioned obedience of policy-executers [27]. In fact, bakers can play a role in implementing the policy of salt reduction in bread both as facilitator and deterrence to achieving the goals of this policy. Engaging bakers in the policy of salt reduction in bread, a commitment can be further built in these individuals to better implement the policy and to make the best use of their practical experiences in more rational policies [26]. Considering the role of salt in bread preparation as a controller of yeast fermentation [28] and given the direct effect of ambient temperature on this function, it can be concluded that the amount of salt used in bread depends on the temperature of the environment as well. With regard to the geography of Iran and difference in temperature between the coldest and hottest regions, it is necessary to determine the standard salt content in bread according to weather conditions of different areas instead of formulating a single salt standard in bread.

It is notable that most of the countries that have successfully reduced salt in bread, including the United Kingdom [24], Finland [29], and New Zealand [30] have a small area, which can be considered as a factor involved in successful implementation of the policy of salt reduction in bread. In some other countries, such as Kuwait [31], almost all flour and bread are produced and distributed by the government. Therefore, being a policy-maker and a policy-executer in this area at the same time is an important factor affecting successful implementation of the policy of salt reduction in bread.

## 6. Conclusion

Reduction of salt in bread has faced serious barriers especially in its implementation in Iran. Even as this study mainly discussed use of salt in bread in Iran and given the implementation of the mentioned policy and lack of similar investigations on the implementation method of this policy, the present study can be utilized as a model for several countries with similar or different contexts.

## References

[1] Jafari M, Mohammadi M, Ghazizadeh H (2016). Feasibility and outcome of reducing salt in bread: a community trial in Southern Iran. Glob J Health Sci.

[2] Silow C, Axel C, Zannini E (2016). Current status of salt reduction in bread and bakery products—a review. J Cereal Sci.

[3] Israr T, Rakha A, Sohail M (2016). Salt reduction in baked products: Strategies and constraints. Trends Food Sci Tech.

[4] Lynch EJ, Dal Bello F, Sheehan EM (2009). Fundamental studies on the reduction of salt on dough and bread characteristics. Food Res Intl.

[5] Antúnez L, Giménez A, Ares G (2016). A consumer-based approach to salt reduction: Case study with bread. Food Res Intl.

[6] Karizaki VM (2017). Ethnic and traditional Iranian breads: different types, and historical and cultural aspects. J Ethn Food.

[7] Belz MC, Ryan LA, Arendt EK (2012). The impact of salt reduction in bread: a review. Crit Rev Food Sci Nutr.

[8] Tomonari T, Fukuda M, Miura T (2011). Is salt intake an independent risk factor of stroke mortality? Demographic analysis by regions in Japan. J Am Soc Hypertens.

[9] Pasqualone A, Caponio F, Pagani MA (2019). Effect of salt reduction on quality and acceptability of durum wheat bread. Food Chem.

[10] Avolio AP, Clyde KM, Beard TC (1986). Improved arterial distensibility in normotensive subjects on a low salt diet. Arteriosclerosis.

[11] D'Elia L, Rossi G, Ippolito R (2012). Habitual salt intake and risk of gastric cancer: a meta-analysis of prospective studies. Clin Nutr.

[12] Quilez J, Salas-Salvado J (2012). Salt in bread in Europe: potential benefits of reduction. Nutr Rev.

[13] Ghazizadeh-Hashemi SH, Larijani B (2015). National action plan for prevention and control of non communicable diseases and the related risk factors in the Islamic Republic of Iran, 2015–2025. Tehran, Iran: Aftab e Andisheh Publ.

[14] World Health Organization (2011). Global status report on noncommunicable diseases 2010: Geneva. https://www.who.int/nmh/publications/ncd_report2010/en/.

[15] World Health Organization (2010). Creating an enabling environment for population-based salt reduction strategies: report of a joint technical meeting held by WHO and the Food Standards Agency, United Kingdom, July 2010. https://apps.who.int/iris/handle/10665/44474.

[16] Shahram Rafieifar M, Hossein Kazemeini M (2016). Strategies and opportunities ahead to reduce salt intake. Arc Iran Med.

[17] Alireza Khosravi M, Reza Malekzadeh M, Sarrafzadegan N (2012). Advocacy strategies and action plans for reducing salt intake in Iran. Arc Iran Med.

[18] Avramenko N, Tyler R, Scanlon M (2018). The chemistry of bread making: The role of salt to ensure optimal functionality of its constituents. Food Rev Intl.

[19] Buse K, Dickinson C, Gilson L (2009). How can the analysis of power and process in policy-making improve health outcomes?. Off J Int Hosp Fed.

[20] Miles MB, Huberman AM, Huberman MA (1994). Qualitative data analysis: An expanded sourcebook.

[21] Walt G, Shiffman J, Schneider H (2008). ‘Doing’ health policy analysis: methodological and conceptual reflections and challenges. Health Policy Plan.

[22] Walt G (1994). Health policy: an introduction to process and power. https://repository.library.georgetown.edu/handle/10822/870131.

[23] Fontanet I, Davidou S, Dacremont C (1997). Effect of water on the mechanical behaviour of extruded flat bread. J Cereal Sci.

[24] Brinsden HC, He FJ, Jenner KH (2013). Surveys of the salt content in UK bread: progress made and further reductions possible. BMJ open.

[25] Amid J (2007). The dilemma of cheap food and self-sufficiency: The case of wheat in Iran. Food Policy.

[26] Moynihan DP (2006). Ambiguity in policy lessons: The agencification experience. Public Adm.

[27] Walker L, Gilson L (2004). ‘We are bitter but we are satisfied’: nurses as street-level bureaucrats in South Africa. Soc Sci Med.

[28] Kilcast D, Angus F (2007). Reducing salt in foods: Practical strategies.

[29] Salovaara H (2009). Technologies of salt reduction in bread: issues, problems and solutions. Salt in bread: technical, taste and other parameters for healthy eating: CCAB Seminar Brussels, Belgium: Centre de Conferences Albert Borschette (CAAB).

[30] Dunford EK, Eyles H, Ni Mhurchu C (2011). Changes in the sodium content of bread in Australia and New Zealand between 2007 and 2010: implications for policy. Med J Aust.

[31] World Health Organization (2016). The SHAKE technical package for salt reduction. https://apps.who.int/iris/handle/10665/250135.

