# Supplementary material for: Policy analysis of salt reduction in bread in Iran
Source: AIMS Public Health. 2019 Dec 9;6(4):534–45. doi: 10.3934/publichealth.2019.4.534 (PMC6940570; doi:10.3934/publichealth.2019.4.534)
Supplement: Supplementary file 1 [file publichealth-06-04-534-s001.pdf]

AIMS Public Health

AIMS Public Health, 6(4): 534–545.
DOI: 10.3934/publichealth.2019.4.534
Received date: 18 October 2019
Accepted date: 02 December 2019
Published date: 09 December 2019

http://www.aimspress.com/journal/aimsph

Research article

Policy analysis of salt reduction in bread in Iran

Saba Loloei[1], Hamed Pouraram[1], Reza Majdzadeh[2], Amirhossein Takian[3,4], Massomeh Goshtaei[5] and Abolghasem Djazayery[1,*]

[1] Department of Community Nutrition, School of Nutritional Sciences and Dietetics, Tehran University of Medical Sciences, Tehran, Iran
[2] Department of Epidemiology and Biostatistics, School of Public Health, Tehran University of Medical Sciences, Tehran, Iran
[3] Department of Health Services Management and Economics, School of Public Health, Tehran University of Medical Sciences, Tehran, Iran
[4] Health Equity Research Center, Tehran University of Medical Sciences, Tehran, Iran
[5] Iran University of Medical Sciences, Tehran, Iran

* **Correspondence:** Email: djazaiers@tums.ac.ir; Tel: 009821-88955975; Fax: 009821-88955975.

Appendix 1

The matic interview guide
Date:
First name and last name:
Organizational position:
Executive record or length of familiarity with the subject of the project:

1. Do you think reducing salt in bread will help reduce salt intake? What is your suggestion for reducing the salt intake of the Iranian people?
2. What policies have been adopted so far to reduce salt in bread?
3. Do you think the policies for reducing salt in bread have been successful in expressing policy goals and expressing specific measures to achieve these goals? (Content)
4. To what extent the policies salt reduction is clear and understandable for you?
5. If you had the full power of policy making, what did you do to increase the efficiency of salt reduction policies in bread?

6. How do you see the underlying factors (political, economic, social and cultural factors at the national or international level) in formulating and implementing politics?
7. What do you think about the import policies or the self-sufficiency of wheat and their effect on the policies for reducing salt in bread?
8. Is there a sufficient infrastructure (equipment, financial resources, informed human resources, etc.) to implement the existing policies for reducing salt in the country?
9. What do you think of the basic problem in implementing the policy of reducing salt in bread?
10. Do you think the traditional or industrial process of bread production is effective in implementing the policy of reducing salt in bread? Please explain.
11. Do you think the existing policies for reducing salt in bread have been accepted by the general public (please mention the dimensions that need to be changed).
12. Do you think that the existing policies for reducing salt in bread have been accepted by bakers? (Please mention the dimensions that need to be changed)
13. Have you received feedback from other stakeholders (policymakers, policy administrators, or people) regarding the implementation of the policy of reducing salt in bread? please explain.
14. Who are the stakeholders in policy and implementation of salt reduction policies in bread and how do they participate?

At the end, if there's something in the policy of reducing salt in the bread you are interested in referring to.

AIMS Press

© 2019 the Author(s), licensee AIMS Press. This is an open access article distributed under the terms of the Creative Commons Attribution License (http://creativecommons.org/licenses/by/4.0)
